# Rivers of Tears – Convergent, Multi‐Scale Approaches to Monitor and Optimize the Health of Our World's Inhabitants

**DOI:** 10.1002/gch2.202500285

**Published:** 2026-01-18

**Authors:** Eric J. Anderson, Vittorio Sansalone, Luke P. Lee, Rebecca Grainger, Sohie Lee, Albert L. Juhasz, Melissa L. Knothe Tate

**Affiliations:** ^1^ Hydrologic Science and Engineering Program Colorado School of Mines Golden Colorado USA; ^2^ Laboratory for Multiscale Modeling and Simulation Université Paris Est Créteil, Université Gustave Eiffel Créteil France; ^3^ Harvard Medical School Boston Massachusetts USA; ^4^ University of California Berkeley California USA; ^5^ University of Otago Wellington Wellington New Zealand; ^6^ Wellesley College Wellesley Massachusetts USA; ^7^ Future Industries Institute University of South Australia Adelaide Australia; ^8^ Blue Mountains World Interdisciplinary Innovation Institute Blue Mountains Australia

**Keywords:** biotechnology, convergence, ecosystems, environment, epidemiology, geospatial approaches, multiscale approaches, nanotechnology, one health, PFAS, toxins

## Abstract

The connectivity and interdependence of our world and its inhabitants’ health have come under increasing focus. Elucidation of the common and interdependent mechanisms of health and disease requires approaches that facilitate understanding of complex systems behavior and probing of both individual and collective system parameters. To this end, multiscale physical and computational modeling offers a particularly powerful tool to predict behavior over vast time and length scales. Other novel technologies, e.g., rapid isolation nanotechnology developed to analyze nanoscale small extracellular vesicles in ocular tears, enable tracking of “fingerprints” from diseases as diverse as ocular to neurodegenerative (e.g., dementia) and cancer. In the future, it will be possible to track the health and disease of ecosystems and their inhabitants, using geospatial and epidemiological approaches, as well as novel biotechnologies, to prevent and mitigate disease processes and enhance well‐being. These concepts are applied by way of an exemplary approach to understand and address the impact of toxic, recalcitrant manmade chemicals (i.e., PFAS) on the health of ecosystems and their diverse inhabitants. Such convergent efforts will be necessary and a priority for solving the complex problems threatening the health of our planet and its inhabitants.

## Graphical abstract

Probing and sustaining the health of Earth's ecosystems and their inhabitants requires convergent approaches at multiple length and time scales. Predictive computational and physical modeling, together with novel biotechnologies applied in unexpected ways, are paving the way for discoveries at the intersection of epidemiology and geospatial science, with implications for the health of our planet and all its inhabitants.

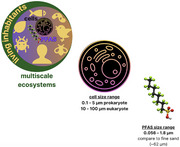

## 1 Introduction

Nearly sixty years ago, an iconic line from the 1967 film The Graduate presaged a materials revolution – “Plastics…there's a great future in plastics….” – Dustin Hoffman as “the graduate” is advised upon graduation from college, as if being let in on a precious secret, a path to future success. Little did we know that within half a century, the molecular components of the pervasive plastics wrapping our food, bottling our drinking water, making up our personal care products, and even protecting our bodies (medical devices including surgical meshes, catheters, inhalers, etc.) and our habitats (firefighting foams) [1], would be taken up by and integrated into our physiological processes. This is detrimental to our health: polyfluoroalkyl substances (PFAS) are a family of synthetic chemicals used in plastics and coatings, can accumulate and persist in the environment and human body, and appear to have manifold deleterious health effects (Table 1) [1]. While we have a detailed understanding of elemental toxins of relevance, our understanding of PFAS toxicities is in its nascency. As such, PFAS provides contemporary lessons for other current and future toxins. Perhaps the “future in plastics” is not so great after all.

**TABLE 1:** Prognostic potential for future toxins via historical and contemporary case studies.

Substance/material toxicity	Time scales		Length scales	
	Persistence times	Historical | human context	Maximal dispersal	Smallest unit material length scale
Lead (element Pb) [2, 3], a naturally occurring metal, remains indefinitely in soil and water once released into the environment – all but a minor amount found in the environment is the result of human activities. Neurotoxin Affects every system in the human body, but neurotoxicity is the most devastating effect for humans.	Atmosphere Lead particles and dust can remain in the air for circa 10 days before settling Environment (soil, water) remains forever unless remobilized or removed; it binds strongly to soil and does not degrade Blood ∼ 1 month Soft Tissues ∼1–1.5 months Brain ∼2–3 years Bone ∼25–30 years	Used as early as 6400 B.C. in decorative objects [3, 4] “[S]now falling at the time of the Ancient Greeks and Romans contained an unexpectedly high concentration of lead.” [4, 5] “[Higher than expected levels of lead pollution” during the Middle Ages, from around 1000–1500 AD [5]. “The amount of lead precipitated from the atmosphere between 500 BC and 300 AD totalled 15 per cent of the lead pollution caused this century by leaded petrol” [5] – Since 2021, lead has no longer used in fuel/petrol.	Water – from source (typically pipes) to faucet tests, circa 14–15 m Dust, airborne particles – hundreds of meters, depending on wind, decreasing with wet conditions [6]	0.1–10 microns (lead dust)
Arsenic (element As) [3, 7, 8] Highly toxic in inorganic form [9] Carcinogen Neurotoxin Long‐term – effects: every system in the human body ‐cardiovascular disease ‐diabetes	Environment Deriving from natural and anthropogenic sources (metal mining, smelting) [8] Persists up to 8000 years [9] Of note: Greatest risk of poisoning due to contaminated water; naturally present in the groundwater of several countries [9] Blood Half‐life of 2–10 h Tissues Hours Keratin‐containing tissues (skin, hair, nails) Months – years	Discovered 1250 AD [3] Has been used historically as a poison (without taste, smell, colour), a chemotherapy agent, wood treatment Currently used for the manufacture of electronics, hardening of lead alloys	Similar to lead	0.05 micron
PFAS [1, 10] Immunotoxin Endocrine disruptor Metabolic disorders Carcinogen	Environment (soil, water) remains for decades to thousands of years; can enter the environment at any stage of their lifecycle, from manufacture to disposal Human body Half‐life from 2 years to decades, depending on the compound	Discovered in the 1930s, with widespread use since the 1950s More than 15,000 known PFAS‐containing compounds Highly resistant to chemical, physical, and biological degradation	1000s of km in air and water, via dust and sea spray [10]	0.056–3 microns “Short‐chain” PFAS, containing fewer than eight carbons, and “ultrashort‐chain” PFAAs, with just two to three carbon atoms
Contemporary and “Future toxins” e.g. glyphosate – other emerging toxins not yet identified or characterized e.g., Glyphosate has been identified as a neurotoxin [11] and a potential carcinogen by the International Agency for Research on Cancer and is still under study.	Currently under study	Glyphosate is the main chemical component of Round‐Up and other products, a widely used weedkiller (herbicide)	Currently under study	Currently under study

In the following, we address current challenges in detecting, understanding, and mitigating the effects of PFAS as a case study for the age‐old, yet perpetual, unsolved problem of toxin contamination of the environment and its inhabitants. Compared to inorganic contaminants (Table 1) including lead (Pb), arsenic (As), mercury (Hg, used as early as 1500 B.C.), chromium (Cr, discovered 1797), and cadmium (Cd, discovered 1817) [3], where toxicity has been recognized for centuries, PFAS of which there are more than 15 000 compounds, have come under scrutiny only recently and are, comparatively, poorly understood. In using PFAS as a case study, we emphasize the imperative to take interdisciplinary, innovative approaches, bridging both length and time scales, to predict and prevent dispersal of toxins detrimental to human and environmental health, and with unknown future ramifications. We hypothesize that not only are we what we eat, but also, we are what we live; we are our environment. We explain that our environment reflects us and our actions as individuals, which scales up to the health of our ecosystems and our planet. The intersection of human and environmental health, and the ubiquitous connectivity of life and physiological processes, manifests from nano to meso scales and from millisecond cellular signaling processes to millennia of evolution.

## 2 Connectivity & Health, from Human to Ecosystems, and Methodologies to Bridge Them

**FIGURE 1:**
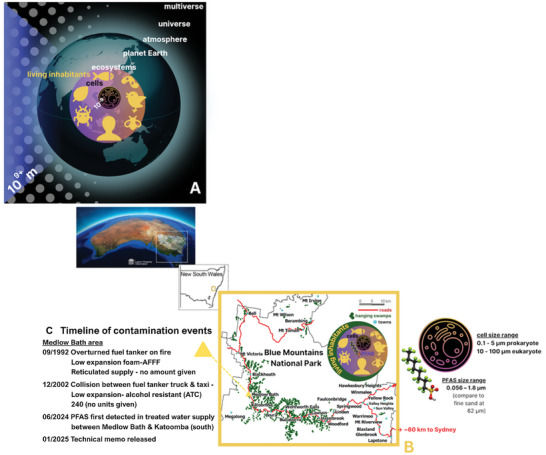
Top‐down and bottom‐up, multiscale impact of (and on) inhabitants of Earth's ecosystems. (A) OneHealth encompasses the health and well‐being of our world and its inhabitants (animals, plants, insects, etc.). These worlds encompass the multiverse (infinite galaxies), the universe, planets (atmosphere and planet Earth), and ecosystems. Of note, animals, plants, insects, etc., themselves are built and inhabited by cells [13]. The observable universe expands beyond 8.835 × 10^23^ km or on the order of about 10^27^ m. Subcellular organelles, some bacteria, as well as chemicals such as per‐ and polyfluoroalkyl substances (PFAS, also known as “forever chemicals”), are on the length scale of nanometers (10^−9^ m, see B). Current OneHealth initiatives focus on the health and well‐being of the Earth and its inhabitants. (B) The effect of this connectivity, between our worlds and their inhabitants, on the flora and fauna of diverse ecosystems and organisms around the globe. Here, an example is depicted with the spread of human‐produced PFAS materials through the freshwater supply of the greater Sydney (Australia) water catchment. The hanging swamps of the World Heritage‐listed Blue Mountains National Park in New South Wales (NSW) represent the local ecosystem. Plants native to hanging swamps have been shown to exhibit unique PFAS filtering capacity [14]. The hanging swamps serve as water sequestering and storage zones for human drinking water, e.g., in the greater Sydney water catchment of the Blue Mountains [16]. Map adapted from original with permission. (C) Timeline of PFAS detection and ongoing discoveries in the Blue Mountains water catchment of Sydney, Australia. Identified contamination sites near Medlow Bath are identified in B (yellow triangle) [16].

Physiology and health are ever‐changing, in space and time. Like a storm gathering on the horizon, the onset and progression of human diseases are just as challenging to predict accurately as they are to alleviate or reverse. Since the COVID‐19 (coronavirus 2019) pandemic [12], the connectivity and interdependence of our world's and its inhabitants’ health have come under increasing focus, providing the impetus for the 2021 formation of the Blue Mountains World Interdisciplinary Innovation Institute, dedicated to the health and well‐being of our world's inhabitants (Figure 1) [15]. Similarly, the “One Health initiative” regained new momentum during the pandemic. Defined as “an integrated, unifying approach that aims to sustainably balance and optimize the health of people, animals, and ecosystems,” One Health “recognizes the health of humans, domestic and wild animals, plants, and the wider environment (including ecosystems)” as “closely linked and interdependent.” [17, 18, 19, 20, 21].

Deciphering the common and interdependent mechanisms of health and disease, across the numerous lengths and time scales relevant to our world – from the Earth to its ecosystems and inhabitants to their resident cells [13] (Figure 1A,B) – requires approaches and methodologies that facilitate the elucidation of complex systems behavior (Table 2) [21, 22, 23]. These approaches enable the probing of system parameters both individually and collectively, to identify which combination of spatiotemporal (in space and in time) variables exerts the most significant effects on outcome measures of interest. Multiscale physical and computational modeling offers a particularly powerful tool in this context, because outcomes can be predicted over vast time and length scales. Examples of such modeling include evolutionary changes, which occur over time scales of approximately 10^8^ years for the evolution of vertebrates. Similarly, pathogen dispersal, which varies across distances from 10^−8^ m for viruses to 10^−9^ m for per‐ and polyfluoroalkyl [PFAS] particles (Figure 1B) [16], across the surface of the Earth, with the longest geographic distances around 10^7^ m [21, 31, 32, 33]. Developing these models is imperative and will require concentrated efforts from multiple experts and support from communities impacted by these models.

**TABLE 2:** Examples of methods and approaches applied to the system of interest (PFAS case study, Blue Mountains National Park, NSW) at different length and time scales, including technical tools in the physical, digital, and virtual domains, and applied in situ, in vitro, and in silico. The list is not complete. Use of multiple, different approaches on related models provides not only intersecting insights into ecosystem and human (and other inhabitant species’) health at vastly different length and time scales, but also cross‐validation of model parameters.

System of interest [unknowns]	Method | Approach [unknowns]	Application notes | model type In Situ, in Vitro, in Silico
(A) Freshwater basin, Sydney 3D vegetation mapping, e.g. – Rainforest canopy – Hanging swamp Hydrological mapping – Rivers, creeks – Dams – Sandstone aquifers [24] (unknowns: retention of “old” water vs. new flows from e.g. rain [27]; retention capacities and retention/release rate of water in porous sandstone vs. hanging swamp plants) retention [25, 26] Weather mapping	High penetration airborne LiDAR [26] 3D aquifier visualisations [currently not available for the Blue Mountains NSW] [28] Water assessments, e.g., volumetric capacities [29], release schedules from dams, etc. Streamflow forecasts, 7‐day and season [25] Bureau of Meteorology [30]	In situ In situ In silico
(B) Toxin dispersal mapping Environmental – Water – Soil – Atmosphere Populations – Fauna, e.g., humans, wildlife – Flora – e.g., hanging swamp plants	e.g. PFASPFAS testing of – Water – Soil – Atmosphere – Plants – Blood – Exosomes	In situ In Vitro
(C) Contamination events – Acute (fire control) – Chronic “known” (waste, human sources, etc.) – Chronic unknown	Geospatial mapping of event colocalization	In silico
(D) Health monitoring Ecosystem Human health, e.g. geospatial distribution of: – Cancers – Cardiovascular events – Endocrine Disorders Etc.	Geospatial mapping of event colocalization	In situ In silico
(E) Toxin mitigation, e.g., via Swamp plants – Species endemic to BMNP – Non‐endemic species New technologiesetc.	Testing of PFAS mitigation in endemic vs. Nonendemic plants	In situ In vitro In silico

Other novel technologies will enable disease insights that were previously inconceivable and can have other unexpected applications. For example, rapid isolation nanotechnology used to analyze nanoscale small extracellular vesicles (sEVs, exosomes) in ocular tears allows for unprecedented investigation of the disease “fingerprints”, related to both eye and systemic diseases, including neurodegenerative diseases and cancer. Excitingly, these technologies facilitate the detection of toxins and pathogens in rivers and watersheds. Similarly, a revolution in imaging and computing over the past decade has opened the door for coupled, seamless organ‐to‐molecular scale imaging and modeling as tools for discovery, biotechnology development, and diagnostics [34, 35, 36, 37], as applied to understand the onset and progression of degenerative joint disease, in guinea pig models and humans. Additionally, leveraging geospatial and state‐of‐the‐art computing technologies allows for Google Maps‐like accessibility to study cell population health within the ecosystems of human tissues and organs, akin to epidemiological studies of cell populations in individual patients [19, 20, 38, 39, 40, 41, 42]. Hence, in the not‐too‐distant future, it will be possible to track the health and disease of ecosystems and their inhabitants, paving the way for new approaches and technologies to prevent and mitigate disease processes and enhance well‐being.

A common theme across these paradigm‐changing studies is the implementation of convergent approaches to study complex, interdependent, and often intractable problems. The U.S. National Academies of Science defines convergence as integrating “expertise from life sciences with physical [referring to physics, chemistry, materials science, mathematics, and computational] sciences, medicine, and engineering to form comprehensive synthetic frameworks that merge areas of knowledge from multiple fields to address specific challenges. Convergence builds on fundamental progress made within individual disciplines but represents a new way of thinking about [the research process and the strategies that enable it], as emerging scientific and societal challenges cut across disciplinary boundaries… The concept of convergence … is thus [intended] to capture two dimensions: the convergence of the subsets of expertise necessary to address a set of research problems, and the formation of the web of partnerships involved in supporting such scientific investigations and enabling the resulting advances to be translated into new forms of innovation and …products.” [21] Convergent efforts will be necessary and a priority for solving the complex problems threatening the health of our planet and its inhabitants.

For instance, a comprehensive approach to understanding and addressing the impact of per‐ and polyfluoroalkyl substances (PFAS) and their precursors, from everyday household items used by humans, demands convergent – both interdisciplinary and innovative – strategies. PFAS toxicity represents an urgent and widespread global challenge, from Maine (USA) [43] to the Blue Mountains freshwater catchment of the Sydney (Australia) basin (Figure 1B). “Widespread contamination from [PFAS] has the potential to impose an unsustainable burden on [public] and private resources” [43]. Alarmingly, human exposure to PFAS is known to disrupt the body's natural hormone system, affecting reproduction, development, and metabolism [43], while effects on other species and kingdoms of life remain limited. Identified direct impacts of PFAS are numerous and include the need for water sampling and soil testing, water filtration, study of impacts on flora and fauna, the requirement for safe replacement fire retardant materials, the necessity for blood testing and medical monitoring and associated treatment, as well as public water system treatment.

## 3 PFAS Case Study in the Blue Mountains Water Catchment

The recent (2024/2025) detection of PFAS in the freshwater catchment of the Blue Mountains of New South Wales, Australia (Figure 1B) is noteworthy. A seven‐month investigation identified the source of this contamination to aqueous firefighting foam used in 1992 and 2002 in response to two petrol‐tanker accidents on a main road through the protected environmental area. In 2000, the NSW Rural Fire Service and Fire & Rescue NSW began phasing out the use of PFAS, fully discontinuing use by 2007. Unfortunately, “PFAS chemicals persist in the environment, in this case 32 years after the original contamination event…” and “…any contamination may be moving downstream … via rainfall and surface water runoff”, so potentially exerting adverse environmental and inhabitant health outcomes well into the future [44].

The ongoing global presence of products containing highly mobile PFAS highlights the urgent need for abatement and treatment solutions due to potential negative effects on environmental health. Notably, both aquatic and terrestrial plants have been demonstrated to possess natural filtration capacity for PFAS, and it has been suggested that “constructed floating wetlands” could provide a “passive remediation approach for PFAS‐impacted surface water” [14]. Furthermore, the hanging swamps of the Blue Mountains constitute a crucial aspect of the world heritage‐listed national park ecosystem (Figure 1B). While new solutions such as constructed floating wetlands are compelling, protecting the existing natural resources that perform this filtration capacity will be equally vital.

## 4 Integration of Biomedical and Ecological Data – A Challenge for Materials‐ and Eco‐Scientists

Multiscale epidemiology becomes more compelling as we increasingly recognize the connectivity of our different worlds; such approaches have been applied to understand the outbreak and spread of diseases as diverse as arthritis [23, 53] to COVID‐19 [45]. Just as our diverse cellular populations inhabit specific tissues of our organismal ecosystem, populations of humans and other species inhabit specific ecosystems of our geographical and social worlds. To further probe connections between and amongst different individuals, populations and environments within our interconnected worlds, there is a need for an integrated framework that links molecular, genetic, geospatial, and computational approaches, including fully accessible, integrated databases, “to improve and cultivate the health and wellbeing of our worlds and our worlds’ inhabitants” [15, 23, 46, 47, 48, 49, 50]. Such tools and reference libraries will increase access to and equity, as well as ubiquity in the application of scientific discoveries and knowledge for the good of all. The creation of open‐source, fully integrated databases using common platforms will facilitate the elucidation and correlation of disparate events, in space and time, as well as the prediction and prevention of future toxin dispersal and other events with the potential to cause harm to various life forms.

A 2013–2016 European Project presaged this need, already before the COVID‐19 outbreak: the so‐called MEDMI project, or **M** edical & **E** nvironmental **D** ata **M** ash‐up **I** nfrastructure project, was designed exactly for this purpose, “to connect diverse databases to improve our understanding of the links between climate, environment, and human health”. MEDMI linked and analyzed “complex meteorological, environmental, and epidemiological data, through the use of the emerging data “mash up” field of computational science”. Unfortunately, MEDMI was decommissioned in 2023. At around the same time, research consortia linking Europe, North America, and Australia began to explore cross‐scale seamless imaging methods with geospatial and epidemiological approaches, as well as the Google Maps API to create epidemiological maps charting arthritis, from the subcellular to organ scale of the human hip [23, 53] [19, 20]. Nearly a decade later, there is a need to reinvigorate the underpinnings of the two projects, to fully integrate biomedical and ecological data.

Perhaps most importantly, the time is ripe to challenge chemists, materials,, and eco‐scientists to integrate OneHealth principles together with the Hippocratic Oath to “do no harm” in developing new materials and approaches to cultivate OneHealth across ecosystems and their inhabitants [51]. In 1999, the Student Pugwash Group in the U.S. formulated an oath, “…promis[ing] to work for a better world, where science and technology are used in socially responsible ways,” where students “promise to work for a better world, where science and technology are used in socially responsible ways…and [not to use their] education for any purpose intended to harm human beings or the environment…” In 2017, Hill suggested the inclusion of several elements in a proposed oath for scientists, in an opinion piece to the Public Broadcasting service, including “I will take responsibility for the downstream social implications of my work,” and “I will strive to minimize the harm done to humans, other animals, and the environment in the course of my research.” [52] Surprisingly, to date, some 25 years after the original proposal, the oath has not been adopted universally, as a first step in taking individual and professional responsibility in the field.

## 5 Outlook

Human‐created PFAS are just one example of predictable but overlooked negative impacts of modern human existence on our planet. Currently, only humans have the power to harness convergent approaches to tackle threats to health and enhance the well‐being of all the world's inhabitants. Scientists can remain excited about the future potential of their work, but must commit to avoiding harm. Ultimately, we aim to improve human health and the quality of life for everyone on our planet, while inspiring a new generation of scientists, engineers, and citizens focused on convergence and ecosystem preservation.

## Conflicts of Interest

The authors declare no conflicts of interest.

## Disclosures

The coauthors serve on the Scientific Advisory Board of the Blue Mountains World Interdisciplinary Innovation Institute. They have no conflicts of interest to report regarding the subject of this opinion piece.

## Data Availability

The authors have nothing to report.
